# HER-2-positive primary neuroendocrine neoplasms of the breast with signet ring feature: A case report and review of literature

**DOI:** 10.3389/fonc.2022.1029007

**Published:** 2022-12-12

**Authors:** Yunjin Li, Yi Cao, Xiaoying Wu, Ruijie Liu, Kuansong Wang

**Affiliations:** ^1^Department of Pathology, School of Basic Medical Sciences, Central South University, Changsha, Hunan, China; ^2^Department of Pathology, Xiangya Hospital, Central South University, Changsha, Hunan, China

**Keywords:** neuroendocrine neoplasm, signet ring feature, breast cancer, HER-2 positive, review

## Abstract

**Background:**

Primary neuroendocrine neoplasm of the breast (BNEN) is an uncommon breast neoplasm, and in most cases, it presents as hormone receptors positive and HER-2 negative. Moreover, in neuroendocrine neoplasms (NENs), the signet ring feature is a rare morphological subtype, and only a few cases have been reported. Here, we report the case of a primary breast neuroendocrine neoplasm with an unusual signet ring cell appearance in this paper. The documentation of this case, combined with a review of the literature, may add to existing knowledge about the outcome and management of this rare tumor.

**Methods:**

In the present review, we describe a unique case of HER-2-positive primary BNEN with a signet ring feature that has not been reported in English. Additionally, we performed a literature search of the PubMed and Web of Science databases and calculated statistics for clinical data and follow-up.

**Results:**

Our literature search, excluding non-English literature, identified 15 articles with data from 24 cases, including ours. The mean age was 51.25 years (range, 30–79 years), and there were 13 male patients (54%) and 11 female patients (46%). Of the 24 cases, some cases (11/24) were associated with lymph node metastases, a few cases (6/24) had distant metastasis, and the vast majority of cases (23/24) occurred in the digestive system. Primary hepatic signet ring cell neuroendocrine tumor showed slow progression and good prognosis. Lymph node involvement was identified in one of eight (12.5%) documented cases, and one of eight (12.5%) reported cases presented with distant metastatic disease. However, the prognosis of neuroendocrine tumors with signet ring cells in the pancreas and stomach was poor. Lymph node involvement was identified in 9 of 15 (60%) documented cases, and 5 of 15 (33.3%) reported cases presented with distant metastatic disease.

**Conclusion:**

NENs with a signet ring feature is uncommon, and this is the first case report of its occurrence in the breast. Current knowledge is limited to anecdotal experience based on case reports and small case series. We provide a literature review to summarize knowledge about this rare entity.

## Introduction

BNEN is a rare subtype of NENs, accounting for <1%. Gene expression profile analysis has shown that it belongs to the luminal type, which is positive for hormone receptors but mostly negative for HER-2 (1). In addition, NENs with the signet ring feature is extremely rare, and current reports focus on the digestive system. They are characterized by distinct intracytoplasmic vacuoles resembling signet ring cells, which are mucin negative and represent the accumulation of intermediate filaments under electron microscopy (2). The unique histological features may cause difficulty in diagnosis and delay in patient care. Here, we report a case of an incidentally identified HER-2-positive primary BNEN with a signet ring feature and provide a literature review.

## Methods

We identified and reported a rare HER-2-positive BNEN with a signet ring feature; provided case-related information on diagnosis, treatment, and prognosis; and explained our view of the disease. At the same time, we searched the PubMed and the Web of Science to identify related articles published between 1991 and 2021 (2–16). The literature overview identified common patient characteristics, clinical presentations, and patient outcomes, while the data described 24 cases of NENs with the signet feature (**Table 1**).

**Table 1:** Clinical summary of neuroendocrine neoplasms with signet ring feature.

	Author/year	Sex/age	Site	Presentation	LN	Other metastatic sites	Status	Follow-Up
1	Sioutos/1991 (3)	M/68	Liver	8 months enlarging mass	No	No	NED	49 months
2	Aoki/1992 (4)	F/39	Liver	6 months hypochondrial mass	No	No	NED	14 months
3	Young/1998 (5)	M/48	Liver	8 years increasing abdominal fullness	No	No	NED	5 months
4	Lau/2006 (6)	F/35	Liver	6 months abdominal pain	No	No	NED	60 months
		M/41	Liver	a mass following ultrasound examination	No	No	NED	8 months
5	Zhu/2010 (2)	M/49	Liver	a mass following CT examination	No	No	NED	10 months
6	Haq S/2015 (7)	F/43	Liver	2 years low grade fever, weight loss and intermittent epigastric pain	No	Yes	AWD	18 months
7	Bansal/2021 (8)	F/47	Liver	2 years hypochondrium dull aching pain	Yes	No	AWD	14 months
8	Stokes/1998 (9)	M/30	Pancreas	1-year abdominal pain	NS	Yes	Re	24 months
9	Montiel/2003 (10)	M/37	Pancreas	acute pancreatitis; recurrent gastric ulcers	Yes	Yes	NED	24 months
		F/55	Pancreas	Biliary obstruction	No	Yes	DOD	1 months
		M/58	Pancreas	Biliary obstruction	No	No	NED	48 months
		F/79	Pancreas	Abdominal pain	No	No	NED	60 months
10	Chetty/2004 (11)	M/50	Pancreas	Acute abdominal pain	Yes	No	NS	NS
11	Shia/2004 (12)	M/60	Pancreas	A pancreatic mass and multiple bilobar liver nodules on abdominal ultrasound and CT scan.	No	Yes	DOD	73 months
12	Serra/2006 (13)	M/35	Pancreas	NS	Yes	No	NED	24 months
		M/55	Pancreas	NS	Yes	No	NED	60 months
		F/45	Pancreas	NS	No	No	NS	NS
		F/41	Pancreas	NS	Yes	No	NS	NS
		F/67	Pancreas	NS	Yes	No	DOD	24 months
13	Miyazaki/2018 (14)	M/53	Pancreas	Two pancreatic head masses and a liver nodule on CT scan	Yes	Yes	NED	54 months
14	Morii/1999 (15)	F/69	Stomach	3 months anorexia and body weight loss	Yes	Yes	DOD	7 months
15	Sugihara/2004 (16)	M/72	Stomach	Diagnosed by endoscopic examination	Yes	No	NS	NS
16	Current	F/54	Breast	A lump on left breast during self-examination	Yes	No	NED	9 months

LN, lymph nodes; NED, no evidence of disease; AWD, alive with disease; DOD, died of disease; Re, recurrence; NS, not stated.

## Case report

**Figure 1:**
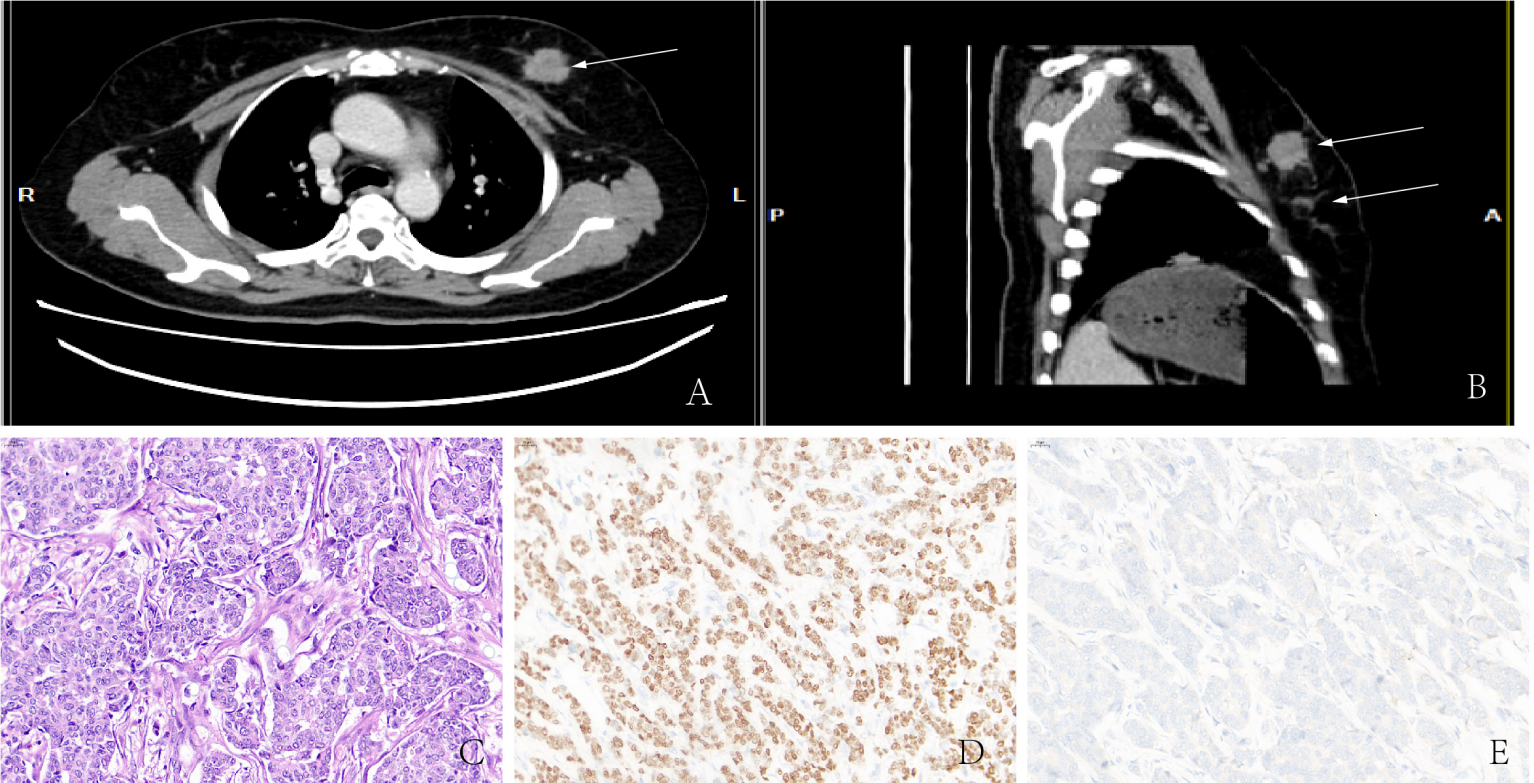
**(A, B)** CT showed high-density masses located in the lateral upper quadrant of the left breast. **(C)** Non-special type of invasive breast cancer. Immunohistochemistry showed that malignant cells are positive for ER **(D)** but negative for Syn **(E)**.

**Figure 2:**
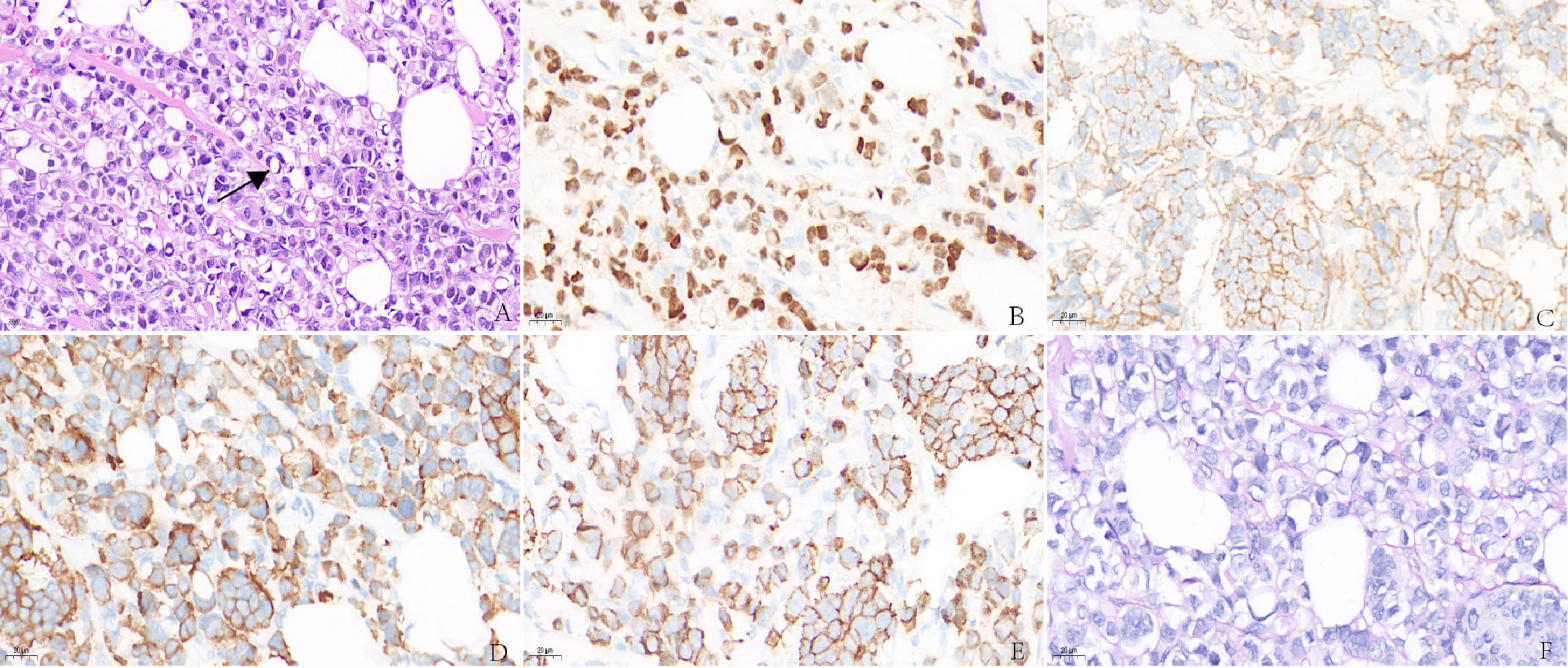
**(A)** Invasive carcinoma of the breast with signet ring feature. Immunohistochemistry showed abnormal cells are positive for ER **(B)**, HER-2 **(C)**, Syn **(D)**, E-cadherin **(E)**, but negative for PAS **(F)**.

A 54-year-old woman discovered two lumps on her left breast during a self-examination. She was diagnosed with “left breast invasive cancer” by McMotong puncture in another hospital and was admitted to our hospital in July 2021. In her physical examination, a 3.5×3 cm firm lump and a 2.5×2 cm firm lump with no pain or nipple discharge were palpated in the upper outer quadrant of the left breast. A 0.5×1 cm enlarged lymph node was palpable in the left axilla. Ultrasonography revealed ill-defined nodules of hypoechoic areas in the left breast and some with variably echogenic intensity in the left lymph node. Computed tomography (CT) scans showed that nodules focus in the left breast with blurred margins and marked enhancement (**Figures 1A, B****)**. Metastatic diseases were excluded with CT examinations in the thorax, abdomen, brain, and other further investigations, which found no more positive signs. The patient underwent radical left mastectomy with axillary lymph node dissection. Macroscopically, one resected tumor at 12 o’clock from the nipple 5 cm was a 3.0 cm × 1.8 cm × 1.5 cm gray–white firm mass (tumor 1) and another resected tumor at 3 o’clock from the nipple 6.5 cm was a 1.6 cm × 1.0 cm × 0.4 cm gray–yellow firm mass (tumor 2). Microscopically, tumor 1 was a non-special type of invasive breast cancer, histologically grade 2 (**Figure 1C**). Neuroendocrine differentiation can be observed in tumor 2, which consisted of medium size cells with eosinophilic cytoplasm separated by fibers and arranged into flakes and nests. Meanwhile, many tumor cells contained eccentric, clear paranuclear vacuoles resembling signet ring cells (**Figure 2A**). A small amount of ductal carcinoma *in situ* can be seen under the microscope in tumor 2. Metastases were seen in lymph nodes (5/21), and isolated cancer cells were seen in one lymph node. The pathological staging was pT2N2Mx.

**Figure 3:**
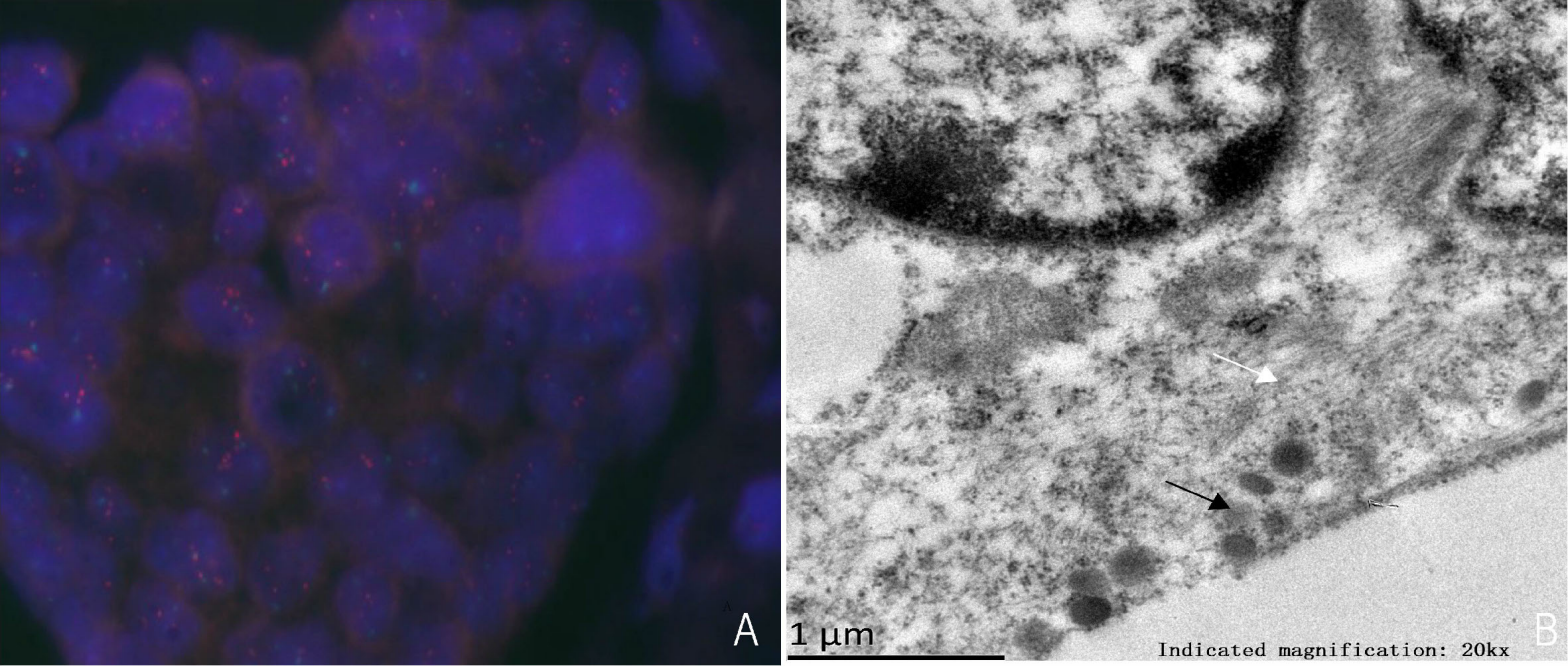
**(A)** HER-2 detection by FISH, HER-2/CEP17>2.0. **(B)** Electron microscopy shows aggregation of numerous neurosecretory granules (black arrows) and intermediate filaments (white arrows).

Immunohistochemistry in tumor 1 showed that these malignant cells were positive for estrogen receptor (ER) but negative for synaptophysin (Syn) (**Figures 1D, E****)**. At the same time, immunohistochemistry in tumor 2 showed that these malignant cells were positive for ER (**Figure 2B**) and GATA binding protein 3 (GATA-3) but negative for epidermal growth factor receptor (EGFR) and cytokeratin 5/6 (CK5/6). HER-2 status was 2+ (**Figure 2C**) and fluorescence *in situ* hybridization (FISH) demonstrated heterogeneity in HER-2 gene amplification in this tumor tissue (**Figure 3A**). Syn and E-cadherin immunopositivity were on the tumor membranes (**Figures 2D, E****)**. Special staining for periodic acid–Schiff stain (PAS) was negative (**Figure 2F**). Electron microscopy confirmed that the neurosecretory granules in the cytoplasm were clustered or locally distributed with intermediate filament aggregation (**Figure 3B**). After the operation, this patient received dose dense AC (four cycles) followed by paclitaxel in combination with trastuzumab and pertuzumab for 3 months, then trastuzumab and pertuzumab for 1 year. Endocrine treatment with an aromatase inhibitor was recommended for 5 years. Her condition remained stable for 9 months without the progression of the primary tumor.

## Results

The average age of NENs with the signet ring feature was 51.25 years old (range, 30–79 years), and the average follow-up time of 24 cases was 29.3 months. Among them, some patients died of the disease, three cases of pancreatic neuroendocrine carcinoma, and one case of gastric neuroendocrine carcinoma. According to the review of the cases, the clinical manifestation of the primary hepatic signet ring cell neuroendocrine tumor was mild, the tumor progressed steadily, and there was no tumor recurrence or metastasis after resection (2–8), but signet ring cell neuroendocrine tumor of the pancreas showed a poor prognosis with the characteristics of high invasiveness, high recurrence rate, and high metastasis rate (9–14). Perhaps, the prognosis is closely related to the malignant degree of the primary tumor.

NENs with the signet ring feature is characterized by eccentric, pale, or eosinophilic intracytoplasmic vacuoles that lead to various terms from ‘‘paranuclear clear zone” (3, 5), ‘‘signet cell” (4, 7–9, 15, 16), to ‘‘rhabdoid” (10–14) according to their light microscopic appearances. These intracytoplasmic inclusions are presumed to be caused by abnormalities in the metabolism and organization of intermediate filament-cytokeratin (3–5, 12). Nonetheless, unlike typical mucus-secreting imprinted cells, they are mucin negative. Electron microscopy revealed that a large number of microfilaments and intermediate filaments are present in this type of NENs, which contains neuroendocrine granules. This ultra-structure explains the crowding of the nucleus to one side and the brightness around the nucleus, which is presumed to be a degenerative change in the tumor.

In 2003, the World Health Organization (WHO) considered mammary neuroendocrine tumors as a separate entity of breast cancer based on the definition provided by Sapino et al. (17). Hence, BNEN were defined as tumors of epithelial origin that resemble gastrointestinal and pulmonary neuroendocrine tumors in morphology, expressing neuroendocrine markers in at least 50% of the total cell population. In 2012, the WHO recognized that the 50% threshold for neuroendocrine marker-expressing cells was unreasonable (18). In the new classification, invasive carcinomas with neuroendocrine differentiation were included in the BNEN group regardless of the percentage of tumor cells expressing neuroendocrine markers. BNEN is an uncommon subtype of NENs, accounting for <1%, and gene expression profile analysis has shown that it belongs to the luminal type, which is positive for hormone receptors but negative for HER-2 in most cases (1). Makretsov et al. have suggested that HER-2 oncoprotein positive is associated with poor prognosis (19). Nonetheless, the prognostic role of anti-HER-2 therapy in BNEN is unclear. Presumably, BNEN is the same as other invasive breast cancers, and a 9-year case follow-up reported that HER-2-positive patients survived disease free for 9 years after treatment with trastuzumab (20). Gevorgyan et al. reported that anti-HER2 therapy was shown to be effective in a BNEN patient who relapsed in bone after 30 years (21). Correspondingly, it is reasonable to use anti-HER2 therapy for HER2-positive BNEN. In addition, patients diagnosed with positive hormone receptor BNEN can receive adjuvant endocrine therapy (22). Chemotherapy is also an option as adjuvant therapy in subjects at high risk of recurrence or as neo-adjuvant therapy in those with locally advanced or unsuitable surgical BNEN. It seems reasonable to use the same chemotherapeutic approach applied to ductal breast cancer. Therefore, regimens that include anthracyclines and taxanes should be preferred when chemotherapy is indicated (23). In this case, the patient had two poor prognostic factors, namely, lymph node involvement and positive HER-2, and the AC-TPH regimen was selected according to the APHINITY study (24). After trastuzumab-based adjuvant therapy, following the ExteNET study, we may consider intensive treatment with Neratinib for 1 year (25).

The clinical features of BNEN are indistinguishable from non-specific types of invasive breast cancer, frequently as a palpable lump or abnormal screening program. Therefore, the diagnosis of BNEN is based on histopathology, relying on pathologists to identify specimens with neuroendocrine differentiation and perform immunohistochemical staining for neuroendocrine markers (CgA/B and/or Syn). In addition, the signet ring feature is a rare morphological subtype of NENs, and current reports predominantly focused on the digestive system. Our case is the first neuroendocrine tumor with a signet ring cell feature in the breast. In breast cancer, the differentiation of signet ring cells can be observed in some histological types, such as primary signet ring cell carcinoma, invasive ductal carcinoma, invasive lobular carcinoma, and metastatic breast cancer. BNEN with this morphology requires extensive immunohistochemistry and other auxiliary tests to avoid misdiagnosis, notably in needle biopsy. According to previous reports, in contrast to pancreatic neuroendocrine tumors with signet ring cell features, primary hepatic signet ring cell neuroendocrine tumors have a more indolent clinical presentation, with slow tumor progression and no tumor recurrence or metastasis after resection. Similarly, the survival of patients with NENs varies significantly at different primary sites. NENs in the rectum and appendix have the best prognosis in the United States, with a median survival of 24.6 and 30.0 years, respectively, while pancreatic and pulmonary NENs have a poor prognosis, with a median survival of 3.6 and 5.5 years, respectively (26). NENs in the rectum and appendix in Taiwan have the best prognosis, with 5-year survival rates of 86.0% and 76.2%, respectively. The 5-year survival rates of NENs in the lung, esophagus, and liver were 32.6%, 16.2%, and 9.2%, respectively (27). The prognostic significance of the signet cell component is still largely unknown. Because only a few cases have been reported to date, the behavior of tumors with this signet ring cell component still requires further study.

## Conclusion

This report presents a case of invasive breast carcinoma with a signet ring feature but negative for PAS, ruling out carcinoma with mucin production. E-Cadherin positivity verified ductal differentiation. ER and GATA-3 were most supportive of mammary origin and formed an effective panel for distinguishing primary breast from primary gastrointestinal signet ring tumors. Syn positivity and neuroendocrine granules were shown under the electron microscope, confirming neuroendocrine differentiation. CT examinations in the thorax, abdomen, brain, and other further investigations can effectively rule out metastatic cancer. We consider this signet ring feature with neuroendocrine differentiation as heterogeneity of this invasive ductal carcinoma of the breast. Nevertheless, the prognosis of this pathological type is unclear. Recognizing this unique histological pattern is essential for proper diagnosis and patient care. The signet ring morphology presents significant challenges for pathological diagnosis.

## Author contributions

YL has made substantial contributions to the conception or design of the work and writes the manuscript. YC revises the manuscript. XW provides the diagnosis of electron microscopy. KW and RL provide the case and revise the manuscript critically for important intellectual content. All authors contributed to the article and approved the submitted version.

## Funding

This work was supported by the Natural Science Foundation of China (Grant No. 81972490).

## Conflict of interest

The authors declare that the research was conducted in the absence of any commercial or financial relationships that could be construed as a potential conflict of interest.

## Publisher’s note

All claims expressed in this article are solely those of the authors and do not necessarily represent those of their affiliated organizations, or those of the publisher, the editors and the reviewers. Any product that may be evaluated in this article, or claim that may be made by its manufacturer, is not guaranteed or endorsed by the publisher.

## References

[1] Pareja F D'Alfonso TM. Neuroendocrine neoplasms of the breast: A review focused on the updated world health organization (WHO) 5th edition morphologic classification. Breast J (2020) 26(6):1160–7. doi: 10.1111/tbj.13863 32383258

[2] Zhu H Sun K Ward SC Schwartz M Thung SN Qin L. Primary hepatic signet ring cell neuroendocrine tumor: A case report with literature review. Semin liver disease. (2010) 30(4):422–8. doi: 10.1055/s-0030-1267542 20960381

[3] Sioutos N Virta S Kessimian N. Primary hepatic carcinoid tumor. an electron microscopic and immunohistochemical study. Am J Clin pathology. (1991) 95(2):172–5. doi: 10.1093/ajcp/95.2.172 1704179

[4] Aoki K Sakamoto M Mukai K Kosuge T Takayama T Hiroshashi S. Signet-ring cell carcinoid: a primary hepatic carcinoid tumor with cytoplasmic inclusions comprising of aggregates of keratin. Japanese J Clin Oncol (1992) 22(1):54–9. doi: 10.1093/oxfordjournals.jjco.a039517 1374135

[5] Oh YH Kang GH Kim OJ. Primary hepatic carcinoid tumor with a paranuclear clear zone: a case report. J Korean Med science. (1998) 13(3):317–20. doi: 10.3346/jkms.1998.13.3.317 PMC3054491 9681813

[6] Lau PP Tint KA Tse GM Lui PC. Primary hepatic carcinoid tumours: report of two cases. Pathology. (2006) 38(5):458–61. doi: 10.1080/00313020600922454 17008291

[7] Haq S Batra VV Majumdar K Javed A Agarwal AK Sakhuja P. Signet ring cell neuroendocrine tumor liver with mesenteric metastasis: Description of a rare phenomenon, with literature review. J Cancer Res Ther (2015) 11(3):658. doi: 10.4103/0973-1482.139604 26458661

[8] Bansal N Satapathy B. Primary multifocal cystic signet ring neuroendocrine tumor of liver: a case report. J Liver Cancer. (2021) 21(2):187–93. doi: 10.17998/jlc.2021.09.17 PMC10035681 37383080

[9] Stokes MB Kumar A Symmans WF Scholes JV Melamed J. Pancreatic endocrine tumor with signet ring cell features: a case report with novel ultrastructural observations. Ultrastructural pathology. (1998) 22(2):147–52. doi: 10.3109/01913129809032270 9615384

[10] Perez-Montiel MD Frankel WL Suster S. Neuroendocrine carcinomas of the pancreas with 'Rhabdoid' features. Am J Surg pathology. (2003) 27(5):642–9. doi: 10.1097/00000478-200305000-00007 12717248

[11] Chetty R Asa SL. Pancreatic endocrine tumour with cytoplasmic keratin whorls. is the term "rhabdoid" appropriate? J Clin pathology. (2004) 57(10):1106–10. doi: 10.1136/jcp.2004.018309 PMC1770450 15452172

[12] Shia J Erlandson RA Klimstra DS. Whorls of intermediate filaments with entrapped neurosecretory granules correspond to the "rhabdoid" inclusions seen in pancreatic endocrine neoplasms. Am J Surg pathology. (2004) 28(2):271–3. doi: 10.1097/00000478-200402000-00018 15043320

[13] Serra S Asa SL Chetty R. Intracytoplasmic inclusions (including the so-called "rhabdoid" phenotype) in pancreatic endocrine tumors. Endocrine pathology. (2006) 17(1):75–81. doi: 10.1385/ep:17:1:75 16760583

[14] Miyazaki T Aishima S Fujino M Ozono K Kubo Y Ushijima Y. Neuroendocrine tumor of the pancreas with rhabdoid feature. Virchows Archiv an Int J pathology. (2018) 473(2):247–52. doi: 10.1007/s00428-018-2398-x PMC6096768 29938394

[15] Morii S Oka K Hakozaki H Nihei T Mori N. CEA-producing mucin-negative gastric signet-ring cell carcinoma with neuroendocrine markers: a case report. J Clin gastroenterology. (1999) 29(1):82–5. doi: 10.1097/00004836-199907000-00021 10405240

[16] Sugihara A Nakasho K Yamada N Nakagomi N Tsujimura T Terada N. Neuroendocrine differentiation of periodic-acid schiff and alcian blue-negative signet-ring cell-like cells and tubular adenocarcinoma cells within a gastric cancer. Scandinavian J gastroenterology. (2004) 39(8):795–800. doi: 10.1080/00365520410005775 15513370

[17] Sapino A Papotti M Righi L Cassoni P Chiusa L Bussolati G. Clinical significance of neuroendocrine carcinoma of the breast. Ann Oncol Off J Eur Soc Med Oncol (2001) 12 Suppl 2:S115–7. doi: 10.1093/annonc/12.suppl_2.s115 11762336

[18] Lakhani SR Ellis IO Schnitt SJ Lakhani SA Ellis I Schnitt S. World health organization classification of tumours of the breast. 4th ed. Lyon, France: IARC Press (2012).

[19] Makretsov N Gilks CB Coldman AJ Hayes M Huntsman D. Tissue microarray analysis of neuroendocrine differentiation and its prognostic significance in breast cancer. Hum pathology. (2003) 34(10):1001–8. doi: 10.1053/s0046-8177(03)00411-8 14608533

[20] Marijanović I Kraljević M Buhovac T Karan Križanac D. Rare human epidermal growth factor receptor 2 (HER-2)-Positive neuroendocrine carcinoma of the breast: A case report with 9-year follow-up. Am J Case Rep (2020) 21:e925895. doi: 10.12659/ajcr.925895 33067411 PMC7579747

[21] Gevorgyan A Bregni G Galli G Zanardi E de Braud F Di Cosimo S. HER2-positive neuroendocrine breast cancer: Case report and review of literature. Breast Care (Basel Switzerland). (2016) 11(6):424–6. doi: 10.1159/000453572 PMC5290429 28228710

[22] Alkaied H Harris K Azab B Dai Q. Primary neuroendocrine breast cancer, how much do we know so far? Med Oncol (2012) 29(4):2613–8. doi: 10.1007/s12032-012-0222-z 22467078

[23] Irelli A Sirufo MM Morelli L D'Ugo C Ginaldi L De Martinis M. Neuroendocrine cancer of the breast: A rare entity. J Clin Med (2020) 9(5):1452. doi: 10.3390/jcm9051452 32414120 PMC7291290

[24] Piccart M Procter M Fumagalli D de Azambuja E Clark E Ewer MS. Adjuvant pertuzumab and trastuzumab in early HER2-positive breast cancer in the APHINITY trial: 6 years' follow-up. J Clin Oncol Off J Am Soc Clin Oncol (2021) 39(13):1448–57. doi: 10.1200/jco.20.01204 33539215

[25] Martin M Holmes FA Ejlertsen B Delaloge S Moy B Iwata H. Neratinib after trastuzumab-based adjuvant therapy in HER2-positive breast cancer (ExteNET): 5-year analysis of a randomised, double-blind, placebo-controlled, phase 3 trial. Lancet Oncol (2017) 18(12):1688–700. doi: 10.1016/s1470-2045(17)30717-9 29146401

[26] Dasari A Shen C Halperin D Zhao B Zhou S Xu Y. Trends in the incidence, prevalence, and survival outcomes in patients with neuroendocrine tumors in the united states. JAMA Oncol (2017) 3(10):1335–42. doi: 10.1001/jamaoncol.2017.0589 PMC5824320 28448665

[27] Chang JS Chen LT Shan YS Chu PY Tsai CR Tsai HJ. An updated analysis of the epidemiologic trends of neuroendocrine tumors in Taiwan. Sci Rep (2021) 11(1):7881. doi: 10.1038/s41598-021-86839-2 33846396 PMC8041887

