# Corrigendum: HER-2 positive primary neuroendocrine neoplasms of the breast with signet ring feature: A case report and review of literature

**DOI:** 10.3389/fonc.2023.1154441

**Published:** 2023-02-09

**Authors:** Yunjin Li, Yi Cao, Xiaoying Wu, Ruijie Liu, Kuansong Wang

**Affiliations:** ^1^ Department of Pathology, School of Basic Medical Sciences, Central South University, Changsha, Hunan, China; ^2^ Department of Pathology, Xiangya Hospital, Central South University, Changsha, Hunan, China

**Keywords:** neuroendocrine neoplasm, signet ring feature, breast cancer, HER-2 positive, review

**Incorrect Affiliation**

In the published article, there was an error in affiliation(s) [1]. Instead of “Ruijie Liu^1^ *”, it should be “Ruijie Liu^2^ *”.

The authors apologize for this error and state that this does not change the scientific conclusions of the article in any way. The original article has been updated.

